# Immune Profiling and Rapid Molecular Diagnostics in Severe Respiratory Infections and Sepsis: The Potential Clinical Perspective of CD169 and CD64

**DOI:** 10.3390/microorganisms14092070

**Published:** 2026-09-16

**Authors:** Chiara Cipriani, Marialaura Fanelli, Vita Petrone, Marina Piccari, Emanuela Balestrieri, Claudia Matteucci, Loredana Sarmati, Nicola Cotugno, Marco Iannetta, Antonella Minutolo, Sandro Grelli

**Affiliations:** 1 Department of Experimental Medicine, University of Rome Tor Vergata, 00133 Rome, Italy; chiara.cipriani@uniroma2.it (C.C.); fanellimarialaura@gmail.com (M.F.); vita.petrone01@gmail.com (V.P.); balestrieri@med.uniroma2.it (E.B.); matteucci@med.uniroma2.it (C.M.); grelli@med.uniroma2.it (S.G.); 2 Department of Systems Medicine, University of Rome Tor Vergata, 00133 Rome, Italy; marina.piccari@uniroma2.it (M.P.); sarmati@med.uniroma2.it (L.S.); nicola.cotugno@opbg.net (N.C.); marco.iannetta@uniroma2.it (M.I.); 3 Infectious Diseases Clinic, Tor Vergata University Hospital, 00133 Rome, Italy; 4 Virology Unit, Tor Vergata University Hospital, 00133 Rome, Italy

**Keywords:** CD169, CD64, respiratory infectious diseases, immune profiling, antimicrobial stewardship, early identification

## Abstract

Acute respiratory infections and sepsis remain major causes of morbidity and mortality worldwide. Rapid pathogen identification and characterization of the host immune response are crucial for timely diagnosis and appropriate treatment. CD169 and CD64 have emerged as promising biomarkers for differentiating viral and bacterial infections. This study aimed to characterize hospitalized patients with severe respiratory infections and/or suspected sepsis by evaluating the potential diagnostic performance of CD169 and CD64 and describing pathogen-associated immune profiles. In this pilot study, nineteen patients admitted to Policlinico Tor Vergata were enrolled within the multicenter SIS-NET project together with healthy donors. Respiratory pathogens were identified on sputum and nasopharyngeal swabs using the FAST multiplex molecular assay. Clinical, biochemical, and immunological parameters were collected during hospitalization. Flow cytometry was performed to assess CD169 and CD64 expression and circulating leukocyte subsets. Patients showed marked etiological heterogeneity, including viral, bacterial, fungal, and mixed infections. Elevated inflammatory markers were commonly observed. CD169 expression on HLA-DR+ monocytes significantly increased in patients with only viral infections, especially COVID-19 (n = 4), whereas CD64 expression was higher in patients with only bacterial infections (n = 4). We have also analyzed the opportunistic infections in 5 people that live with HIV infection (PLWH) compared with the other subgroups. These findings support the integration of rapid molecular diagnostics and immune profiling to improve the characterization of severe respiratory infections and highlight CD169 and CD64 as useful biomarkers for patient stratification and clinical management.

## 1. Introduction

In 2011, the World Health Organization (WHO) introduced the first global surveillance case definition for severe acute respiratory infection (SARI). This definition applied across all age groups. The standard definition comprised patients presenting with acute respiratory infection characterized by a history of fever (or measured temperature ≥ 38 °C) and cough. Additionally, symptom onset occurred within the previous 10 days, and the condition required hospitalization [1]. Differential diagnoses encompassed a wide range of community-acquired pathogens. These included respiratory viruses, bacteria, and less common microorganisms. Their distribution varied according to host-related factors such as age, chronic diseases, travel history, and vaccination status. Environmental conditions, epidemiological context, and pathogen-specific characteristics also influenced this distribution.

Respiratory tract infections affected both the upper and lower respiratory tracts. They remained a leading cause of morbidity and mortality worldwide. Lower respiratory tract infections alone were responsible for more than four million deaths annually [2]. Several environmental and non-environmental factors contributed to host susceptibility. These factors included air pollution, overcrowding, tobacco smoke exposure, and poor indoor air quality. Malnutrition, advanced age, chronic comorbidities, immunosuppression, and inadequate immune protection during early life also played a role [3]. Many of these elements impaired immune function. Consequently, they increased the risk and severity of subsequent respiratory infections.

A broad spectrum of viral and bacterial pathogens caused these infections. Their prevalence and clinical significance varied according to host susceptibility, geographic distribution, and epidemiological context. Common viral pathogens included respiratory syncytial virus (RSV), parainfluenza viruses, rhinoviruses, and adenoviruses. Enterovirus D68 (EV-D68), human metapneumovirus, and seasonal influenza viruses were also frequent. Less commonly, respiratory infections resulted from varicella-zoster virus, measles virus, and hantaviruses. Emerging viral pathogens of major public health concern included severe acute respiratory syndrome coronavirus (SARS-CoV), SARS-CoV-2, and Middle East respiratory syndrome coronavirus (MERS-CoV). Novel influenza subtypes and other zoonotic viruses also posed significant threats. In immunocompromised individuals, particularly people living with HIV, cytomegalovirus and herpes simplex viruses caused severe respiratory disease [4].

Bacterial pathogens commonly associated with these infections were *Streptococcus pneumoniae*, *Haemophilus influenzae*, *Moraxella catarrhalis*, and *Legionella pneumophila*. Non-pneumophila *Legionella* species, *Chlamydia pneumoniae*, *Mycoplasma pneumoniae*, *Klebsiella pneumoniae*, and *Staphylococcus aureus* were also frequently identified. Less common bacterial etiologies included *Mycobacterium tuberculosis*, *Burkholderia pseudomallei*, rickettsial infections, and *Coxiella burnetii*. *Leptospira* spp., *Chlamydia psittaci*, *Bordetella pertussis*, and *Salmonella* species were also observed, particularly in high-prevalence geographic regions or in patients with specific risk factors. Multidrug-resistant pathogens should have been considered in patients with recent antimicrobial exposure or other predisposing factors. These pathogens included methicillin-resistant *S. aureus* (MRSA), *Pseudomonas aeruginosa*, *Acinetobacter baumannii*, and extended-spectrum β-lactamase (ESBL)-producing Enterobacterales [4].

In endemic settings, respiratory infections coexisted with other infectious diseases. These included malaria, dengue, chikungunya, tuberculosis, and HIV infection. Such co-infections complicated both diagnosis and clinical management [4].

Respiratory tract infections represented a leading cause of sepsis worldwide and posed a major challenge to the immune system. The progression from a localized pulmonary infection to sepsis depended on pathogen virulence and the host’s dysregulated immune response [5]. Both bacterial and viral pathogens induced an excessive inflammatory reaction. This reaction was characterized by cytokine release, endothelial dysfunction, and organ damage. Ultimately, this process led to sepsis and multiple organ failure [5,6].

The respiratory epithelium and innate immune cells constituted the first line of defense against invading microorganisms. Following pathogen recognition through pattern recognition receptors, the immune system activated inflammatory pathways to promote microbial clearance. However, uncontrolled immune activation resulted in systemic inflammation accompanied by profound immune dysfunction [6]. In bacterial respiratory infections, such as community-acquired pneumonia, the release of pro-inflammatory mediators contributed to endothelial injury and septic progression. These mediators included TNF-α, IL-1β, and IL-6 [7]. Likewise, severe viral infections caused by respiratory viruses induced viral sepsis. This occurred through persistent immune activation, cytokine dysregulation, and coagulation abnormalities. Examples included infections caused by SARS-CoV-2 and influenza viruses [8,9].

Bacterial and viral sepsis shared common immunopathological mechanisms. These mechanisms were characterized by the coexistence of hyperinflammation and immunosuppression. Sepsis was increasingly recognized as a heterogeneous syndrome. In this syndrome, excessive inflammatory responses were accompanied by lymphocyte dysfunction, impaired antigen presentation, and increased susceptibility to secondary infections [6,10]. The lungs played a pivotal role in this process. Pulmonary barrier disruption facilitated both pathogen dissemination and systemic immune activation. This disruption contributed directly to the development of acute respiratory distress syndrome and organ dysfunction [7,8].

A better understanding of the relationship between respiratory infections and immune dysregulation remained essential. It guided the identification of novel biomarkers and targeted therapeutic strategies. These strategies aimed to improve early diagnosis and patient outcomes in both bacterial and viral sepsis [6,10].

Among the emerging host-response biomarkers, CD169 and CD64 gained considerable attention. They served as early indicators of viral [11,12,13] and bacterial infections [13,14], respectively. This utility stemmed from their rapid modulation during immune activation and their potential role in improving the early diagnosis and differential characterization of sepsis [13,15].

Significant knowledge gaps persisted regarding the ability of individual inflammatory biomarkers to reliably rule out bacterial versus viral infections without diagnostic overlap. This limitation was particularly evident within complex or heterogeneous clinical cohorts. The rationale of this study was grounded in the necessity to overcome these diagnostic limitations. To achieve this, we evaluated a dual-biomarker host-response model that leveraged the mechanistically distinct pathways of myeloid cell activation. Consequently, we hypothesized that monocyte CD169 expression served as a highly sensitive and exclusive indicator of acute viral-induced interferon cascades. Conversely, neutrophil CD64 expression functioned as a high-amplitude operational marker specifically driven by bacterial insults. By cross-evaluating the synergistic performance of CD169 and CD64 within our pilot study, we aimed to establish a reciprocal potential diagnostic matrix. Developed within the framework of the SIS-NET project [16], this matrix aimed to robustly differentiate infectious etiologies and mitigate triage delay.

## 2. Materials and Methods

### 2.1. Patient and Healthy Donor Enrollment

Patients (n = 19) with severe respiratory infections and/or suspected sepsis were prospectively enrolled at the Infectious Diseases Unit of the Policlinico Tor Vergata University Hospital in Rome between December 2024 and July 2025. This observational study was conducted within the framework of the project “Severe Infections and Sepsis clinical NEtwork for identification of clinical and diagnostic Markers, immunological monitoring and Target and tailored therapies for adults, children and patients admitted to intensive care units” (SIS-NET, project ID S4-01. P0001).

The study protocol was approved by the local Ethics Committee for the collection and use of human biological samples (Ethical approval: 334.24 CET2 PTV; approval date: 20 December 2024). All participants provided written informed consent for participation in the study and for the collection and use of biological samples, in accordance with the principles of the Declaration of Helsinki (1975, revised in 2013).

Healthy donors (HDs) (n = 19) were recruited at the Blood Transfusion, have been matched for age and sex to the best possible extent with the patients and provided written informed consent. Furthermore, HDs included in the study had no evidence of active inflammatory processes or infectious diseases at the time of sample collection.

At the time of enrollment (T0, corresponding to the beginning of hospitalization and before any kind of treatment), peripheral blood samples, sputum, and nasopharyngeal swabs were collected for the analyses included in this pilot study.

Among the 19 patients enrolled in the study, 4 had SARS-CoV-2 infection alone, 3 had other viral infections, and 4 had bacterial infections only. The remaining 5 patients were HIV-positive and presented with infections of different etiologies. Two additional patients, one with hepatitis B virus (HBV) infection and one with metastatic cancer, were enrolled but were excluded from the analyses performed in this pilot study due to their distinct clinical characteristics.

Patients presenting with coinfections were classified based on clinical and microbiological criteria. Only one patient exhibited a simultaneous viral and bacterial coinfection (influenza and bacterial detection); due to the limited sample size (n = 1), this individual was excluded from subsequent sub-analyses to prevent statistical bias. The remaining coinfected individuals consisted entirely of PLWH. To assess the impact of a significant pre-existing chronic condition on the immune response, these patients were grouped into a single category. This categorization enabled the evaluation of our biomarkers of interest in the context of an important underlying chronic infection during active opportunistic episodes. Therefore, a total of 16 patients were included in the analyses.

To ensure the accuracy of the baseline immunophenotypic profiles and avoid the confounding effects of medical interventions, all peripheral blood samples were systematically collected at the time of hospital admission, strictly prior to the administration of any empirical antimicrobial or antiviral treatments. This approach was crucial since subsequent therapeutic regimens have been shown to significantly modulate and alter the expression dynamics of these targeted biomarkers [17,18].

### 2.2. Blood Sample Collection, Processing and Storage Procedures

Whole blood samples from patients and HD were collected into BD Vacutainer^®^ tubes containing K2EDTA as an anticoagulant. Each sample was processed at the Laboratories of Microbiology and Clinical Microbiology of the Department of Medicine and Surgery, University of Rome “Tor Vergata”. An alphanumeric identification code was assigned to each sample to ensure patient confidentiality and to allow the creation of a dedicated reference database.

All samples were processed according to standardized protocols, following Good Laboratory Practice (GLP) principles.

For each donor/patient, aliquots of whole blood and plasma were stored at −80 °C. Plasma samples were obtained from whole blood after centrifugation at 1600 rpm for 10 min.

### 2.3. Rapid Molecular Identification of Respiratory Pathogens

At the time of hospital admission, sputum and a nasopharyngeal swab were collected from each patient and processed using a rapid molecular diagnostic assay for pathogen identification. The BIOFIRE^®^ FILMARRAY^®^ Pneumonia Panel (PN) Plus is an in vitro diagnostic (IVD) multiplex assay designed to detect clinically relevant respiratory pathogens and antimicrobial resistance (AMR) genes directly from respiratory specimens within approximately 1 h.

The kit used in this study is specifically designed for the analysis of sputum and bronchoalveolar lavage (BAL) samples. In parallel, a nasopharyngeal swab specimen, collected for routine laboratory diagnostic analyses, was also recovered. The analysis of the data related to the identification of the different pathogens yielded identical results in the two specimen types. These findings demonstrate the ability of the kit to detect target pathogens in material obtained by nasopharyngeal swabbing, suggesting its potential applicability to this type of clinical specimen despite its specific intended use for sputum and BAL samples.

Sputum and nasopharyngeal samples were collected using the eSwab^®^ Liquid Amies Elution Swab System (Copan Italia S.P.A., Brescia, Italia), a standardized collection and transport system designed for the recovery and preservation of aerobic and anaerobic bacteria, fastidious microorganisms, viruses and *Chlamydia* species from clinical specimens. Following collection, swabs were immersed in Amies preservation medium, which replaces glycerophosphate with inorganic phosphate and contains calcium and magnesium ions to maintain microbial cellular integrity. This formulation allows the preservation and recovery of fastidious microorganisms, including *Neisseria gonorrhoeae*, *Streptococcus* spp., *Trichomonas* spp., and *Haemophilus* spp.

### 2.4. Flow Cytometry Analysis

Fresh whole blood samples (30 μL) obtained from patients and HD were incubated on ice and protected from light for 20 min with antibodies: anti-CD169 conjugated to phycoerythrin (PE) (clone 7-239), anti-CD64 conjugated to Pacific Blue (PB), and anti-HLA-DR conjugated to allophycocyanin (APC) (clone 22) (Beckman Coulter, Brea, CA, USA). For leukocyte overview, IM Phenotyping Basic Tube (B53309, Beckman Coulter) CD45 (KrO), CD16 (FITC), CD56 (PE), CD19 (ECD), CD14 (PC7), CD4 (APC), CD8 (AF700), CD3 (AF750) were used. After incubation, blood samples were incubated for 15 min in the dark at room temperature with 1.5 mL of VersaLyse Lysing Solution (Beckman Coulter, BC) to lyse red blood cells and select a total leukocyte population. The stained cells were analyzed via CytoFLEX (Beckman Coulter) and CytExpert 2.3 software (BC). CD169 expression was represented as the ratio of CD169 median fluorescence intensity (MFI) between HLA-DR-positive monocytes and lymphocytes (RMFI), as described in previous studies [17,18]. CD64 expression was represented as the ratio of CD64 median fluorescence intensity (MFI) between neutrophils and lymphocytes (RMFI).

For CD64 analysis, granulocytes were identified according to their characteristic high SSC and FSC profile. CD64 fluorescence was quantified within the granulocyte gate without applying an HLA-DR-positive selection. HLA-DR gating was used exclusively for the CD169 analysis.

The gating strategies for CD169 and CD64 RMFI were reported in Supplementary Figure S1, and for the IM Phenotyping Basic was used the analyses shown on the datasheet.

### 2.5. Statistical Analysis

Statistical analysis of groupwise expression levels was performed using the non-parametric Mann–Whitney test for comparisons between two independent samples. Non-parametric effect size analysis (Cliff’s delta) and statistical significance of CD169 and CD64 expression between active infection cohorts and HD. Pairwise associations between continuous variables were assessed using the Spearman correlation coefficient. To account for multiple testing in correlation analyses, *p*-values from Spearman tests were adjusted using the Benjamini–Hochberg false discovery rate (FDR) method, and significance was set at FDR-adjusted *p*-value (q-value) < 0.05. All statistical analyses were conducted using SPSS statistical software (version 23.0 for Windows, Hot Springs, AR, USA).

Correlation analyses were explored because of the small sample sizes within the individual infectious subgroups. Therefore, the observed associations are presented as hypothesis-generating and should not be interpreted as statistically robust or generalizable findings.

## 3. Results

Given the pilot nature of the study and the small sample sizes within the individual clinical subgroups, all between-group comparisons and correlation analyses were considered exploratory. The results are presented as preliminary and hypothesis-generating and should not be interpreted as definitive diagnostic or prognostic evidence. The small subgroup sizes also increase the potential influence of individual observations and limit the ability to account for clinical heterogeneity and potential confounding factors.

### 3.1. Clinical Characteristics, Comorbidities, Respiratory Support, Severity of Respiratory Failure, and Clinical Outcomes

Nineteen patients with severe respiratory infections and/or suspected sepsis were prospectively enrolled in the SISNET project at the Infectious Diseases Unit of the University Hospital Policlinico Tor Vergata (Rome, Italy) between December 2024 and July 2025. The demographic and clinical characteristics of the study cohort are summarized in Table 1 and Table S1. The median age of the cohort was 61 years (range, 31–81 years), and males accounted for 57.9% (11/19) of the enrolled patients.

The most common comorbidities were arterial hypertension (5/19, 26.3%), chronic immunosuppression related primarily to HIV infection (5/19, 26.3%), and chronic respiratory diseases, including asthma and chronic obstructive pulmonary disease (COPD), which were present in 15.8% of patients. Chronic heart disease, diabetes mellitus, and former smoking history were each reported in three patients (15.8%), whereas chronic hepatitis B virus (HBV) infection, metastatic adenocarcinoma, epilepsy, and obesity were each observed in one patient (5.3%). The mean Charlson Comorbidity Index was 3.68, indicating an overall moderate comorbidity burden within the study population.

Respiratory support requirements reflected the heterogeneous clinical severity of the cohort. Venturi mask oxygen therapy was the most frequently administered respiratory support modality (13/19, 68.4%). High-flow nasal cannula (HFNC) was required in four patients (21.1%), whereas non-invasive ventilation (NIV) was used in one patient (5.3%). One patient maintained adequate oxygenation without supplemental respiratory support. Respiratory impairment was assessed using the PaO_2_/FiO_2_ (P/F) ratio. The mean P/F ratio was 248, ranging from 153 to 343. Only two patients (10.5%) presented a P/F ratio above 300, indicating preserved gas exchange. Most patients (13/19, 68.4%) exhibited mild respiratory impairment (P/F 200–300), while four patients (21.1%) had moderate impairment (P/F 100–200). No cases of severe respiratory failure (P/F < 100) were observed.

Overall, the cohort was characterized by predominantly mild-to-moderate impairment of pulmonary oxygenation.

The duration of hospitalization ranged from 2 to 22 days, with a mean length of stay of 7 days. Twelve patients (63.2%) were discharged within the first week of hospitalization, whereas the remaining patients required longer inpatient management. Clinical outcomes were favorable for the entire cohort, as all enrolled patients were discharged alive following completion of their hospital stay.

### 3.2. Microbiological Characteristics of the Study Cohort

The FAST molecular diagnostic assay identified at least one pathogen in all 19 enrolled patients (Table 2), illustrating the marked microbiological heterogeneity of this pilot cohort. Viral, bacterial, and fungal pathogens were detected, and several patients presented with more than one microorganism.

SARS-CoV-2 was identified in four patients (21.1%), while Influenza A virus was detected in four patients (21.1%). Rhinovirus/Enterovirus was identified in three patients (15.8%), whereas Parainfluenza virus, Adenovirus, and Herpes simplex virus type 1 (HSV-1) were each detected in one patient (5.3%).

Among bacterial pathogens, *Streptococcus pneumoniae* was detected in three patients (15.8%), while *Haemophilus influenzae* and *Pseudomonas aeruginosa* were each identified in two patients (10.5%). *Mycoplasma pneumoniae*, *Klebsiella pneumoniae*, *Enterobacter cloacae* complex, and *Staphylococcus aureus* were each detected in one patient (5.3%). Fungal or opportunistic pathogens included *Pneumocystis jirovecii*, detected in two patients (10.5%), and *Candida* spp., detected in one patient (5.3%).

Polymicrobial infections were observed in several individuals. The greatest number of pathogens was detected in patient TSB003, in whom Influenza A virus, *Enterobacter cloacae* complex, *Klebsiella pneumoniae*, and *Pseudomonas aeruginosa* were simultaneously identified. Patient TSB007, who was living with HIV, presented with Rhinovirus/Enterovirus, *Haemophilus influenzae*, and *Streptococcus pneumoniae*. Additional mixed infections were observed in patient TSB010, with HSV-1 and *Pneumocystis jirovecii*, and in patient TSB019, with Influenza A virus and *Streptococcus pneumoniae*.

Five patients (26.3%) were living with HIV and showed heterogeneous clinical and microbiological profiles, including differences in CD4+ T-cell counts, comorbidities, and the presence of concomitant infections. Opportunistic microorganisms, including *Pneumocystis jirovecii* and *Candida* spp., were detected within this subgroup. Because of the substantial clinical heterogeneity among these five individuals, observations regarding immunological and microbiological characteristics of the PLWH group should be interpreted descriptively and cannot be considered representative of the broader population of people living with HIV.

Overall, microbiological findings highlight the heterogeneous composition of this pilot cohort and provide the clinical basis for the exploratory grouping of patients according to their principal infectious condition. However, given the limited overall sample size and the small number of individuals within each subgroup, these groupings were used for exploratory analyses only and should not be interpreted as defining distinct or generalizable immunological profiles.

For subsequent exploratory analyses, the patient with chronic hepatitis B virus (HBV) infection (TSB005) and the patient with metastatic adenocarcinoma (TSB019) were not included in the subgroup comparisons because each represented a unique clinical category within the cohort. Also, TSB003 with viral and bacterial infection was excluded. Their exclusion was intended to avoid the creation of single-patient analytical groups; however, the remaining subgroup sizes were still limited. Therefore, all subgroup comparisons and correlation analyses were considered exploratory and hypothesis-generating.

### 3.3. Assessment of Biochemical Alterations Associated with Renal and Hepatic Function and Systemic Inflammation

The biochemical profile of the enrolled patients was evaluated by comparing individual laboratory values with their corresponding physiological reference ranges (Table 3). Overall, the analysis revealed evidence of systemic inflammation accompanied by mild-to-moderate alterations in liver function, whereas renal function was largely preserved.

Hyperglycemia was observed in 26% of patients, with the highest blood glucose concentration recorded in patient TSB004 (204 mg/dL), diagnosed with Influenza A virus infection. Renal function was assessed by measuring serum urea and creatinine levels. Elevated urea concentrations were detected in patients TSB013, TSB015, and TSB016, with the highest value (101.8 mg/dL) observed in patient TSB016 with SARS-CoV-2 infection. In contrast, serum creatinine levels remained within the normal reference range (0.73–1.18 mg/dL) in most patients, indicating overall preserved renal function despite isolated elevations in blood urea.

Liver function was evaluated through serum albumin and hepatic enzyme measurements. Hypoalbuminemia (albumin < 3.5 g/dL) was identified in several patients, with the lowest concentration (2.47 g/dL) observed in patient TSB017 with SARS-CoV-2 infection. Reduced serum albumin may reflect impaired hepatic protein synthesis, increased vascular permeability, malnutrition, or systemic inflammation.

Evidence of hepatocellular injury was further supported by elevated aminotransferase levels. Aspartate aminotransferase (AST) exceeded the upper reference limit in 63% of patients, whereas alanine aminotransferase (ALT), a more specific marker of hepatocellular damage, was elevated to 15% of the cohort. Conversely, serum alkaline phosphatase (ALP) and total bilirubin concentrations remained within the reference ranges in all evaluated patients. Increased gamma-glutamyl transferase (GGT), suggestive of hepatobiliary involvement, was detected in 15% of patients.

The most pronounced biochemical abnormality was the marked elevation of C-reactive protein (CRP), a well-established biomarker of acute systemic inflammation. More than 90% of patients exhibited CRP concentrations above the normal reference range, with the highest value recorded in patient TSB017 (252.7 mg/L).

Collectively, these findings indicate that the study cohort was characterized by preserved renal function, frequent biochemical evidence of hepatic involvement, and a pronounced systemic inflammatory response, as reflected by the widespread elevation of CRP.

### 3.4. Analysis of Electrolyte Profile, Coagulation Parameters, and Biomarkers of Cellular Damage

Serum electrolyte levels, coagulation kinetics, and systemic biomarkers of cellular damage were systematically analyzed and compared across four distinct patient groups. Group-specific descriptive statistics, expressed as Mean ± Standard Deviation (SD), are detailed in Table 4. Values shifting beyond established clinical reference boundaries have been highlighted to delineate systemic aberrations.

COV group demonstrated a profound impairment in coagulation kinetics. This cohort exhibited a highly suppressed PT ratio (0.74 ± 0.16) and diminished AT activity (0.75 ± 0.15). Concurrently, marked elevations were observed in mean INR (1.27 ± 0.22), aPTT (47.90 ± 2.17 s), and aPTT ratio (1.40 ± 0.00), coupled with hyperfibrinogenemia (493.0 ± 165.6 mg/dL). Electrolyte profiles remained strictly within normal reference thresholds.

VIR group was primarily characterized by borderline hypokalemia (3.43 ± 0.42 mmol/L) alongside clear signs of systemic inflammation and tissue injury. The mean aPTT was mildly prolonged (39.97 ± 3.27 s), while LDH (233.0 ± 15.6 U/L) and plasma fibrinogen levels (843.0 ± 280.0 mg/dL) were notably elevated above baseline values.

The bacteria group exhibited severe electrolyte volatilization, marked by a critical trend toward hypernatremia (145.5 ± 16.8 mmol/L), heavily influenced by patient TSB009 (170 mmol/L). This group also displayed substantial tissue degradation and reactive acute-phase processes, evidenced by prolonged aPTT (40.25 ± 3.75 s), high LDH (340.3 ± 136.4 U/L), and increased fibrinogen (605.0 ± 211.6 mg/dL).

PLWH group presented a combined pattern of consumptive/reactive coagulopathy and prominent cellular damage. Mean INR (1.24 ± 0.38), aPTT (43.04 ± 16.70 s), and aPTT ratio (1.24 ± 0.50) were elevated, reflecting extended clotting times. This cascade occurred alongside hyperfibrinogenemia (595.4 ± 175.6 mg/dL) and the highest absolute levels of cellular destruction observed in the study, marked by severe LDH elevation (363.5 ± 109.6 U/L).

### 3.5. Distribution of Peripheral Immune Cell Subsets Among the Different Infection Groups

The distribution of peripheral immune cell subsets HD, COV, PLWH, patients with other viral infections (VIRUSES), and patients with bacterial infections (BACTERIA) is summarized in Table 5.

Overall, distinct immunophenotypic profiles were observed among the different infection groups. Compared with healthy donors, significant alterations involved both innate and adaptive immune cell populations.

Patients with COVID-199 exhibited a significantly higher proportion of circulating monocytes than healthy donors (*p* < 0.05). Although not reaching statistical significance, this group also showed a tendency toward reduced lymphocyte frequencies and increased granulocyte percentages, consistent with the predominance of innate immune activation during acute SARS-CoV-2 infection.

Similarly, PLWH displayed a significant increase in monocyte frequencies compared with healthy donors (*p* < 0.05). In addition, the proportion of natural killer (NK) cells was significantly higher than in the HD group (*p* < 0.05). PLWH also tended to exhibit increased CD8^+^ T-cell frequencies together with lower CD4^+^ T-cell percentages, reflecting the characteristic immune imbalance associated with chronic HIV infection.

Patients with other viral infections were characterized by a significant expansion of CD8^+^ T lymphocytes compared with healthy donors (*p* < 0.05), whereas the remaining leukocyte subsets were broadly comparable to those observed in the other viral infection groups, despite noticeable interindividual variability.

The bacterial infection group also showed significantly higher CD8^+^ T-cell frequencies than HD (*p* < 0.05). In addition, this group displayed higher proportions of granulocytes and monocytes, together with a trend toward increased CD19^+^ B lymphocytes, suggesting a broader activation of both innate and adaptive immune compartments.

The results of Cliff’s delta analysis was reported in Supplementary Material. Taken together, these findings indicate that the different infectious etiologies are associated with distinct peripheral immune signatures. Increased monocyte frequencies were predominantly observed in COVID-19 and PLWH, whereas expansion of CD8^+^ T lymphocytes characterized both viral and bacterial infections. Increased NK-cell frequencies were a distinctive feature of the PLWH group.

### 3.6. CD169 Expression on HLA-DR^+^ Cells Assessed by Relative Mean Fluorescence Intensity (RMFI)

**Figure 1:**
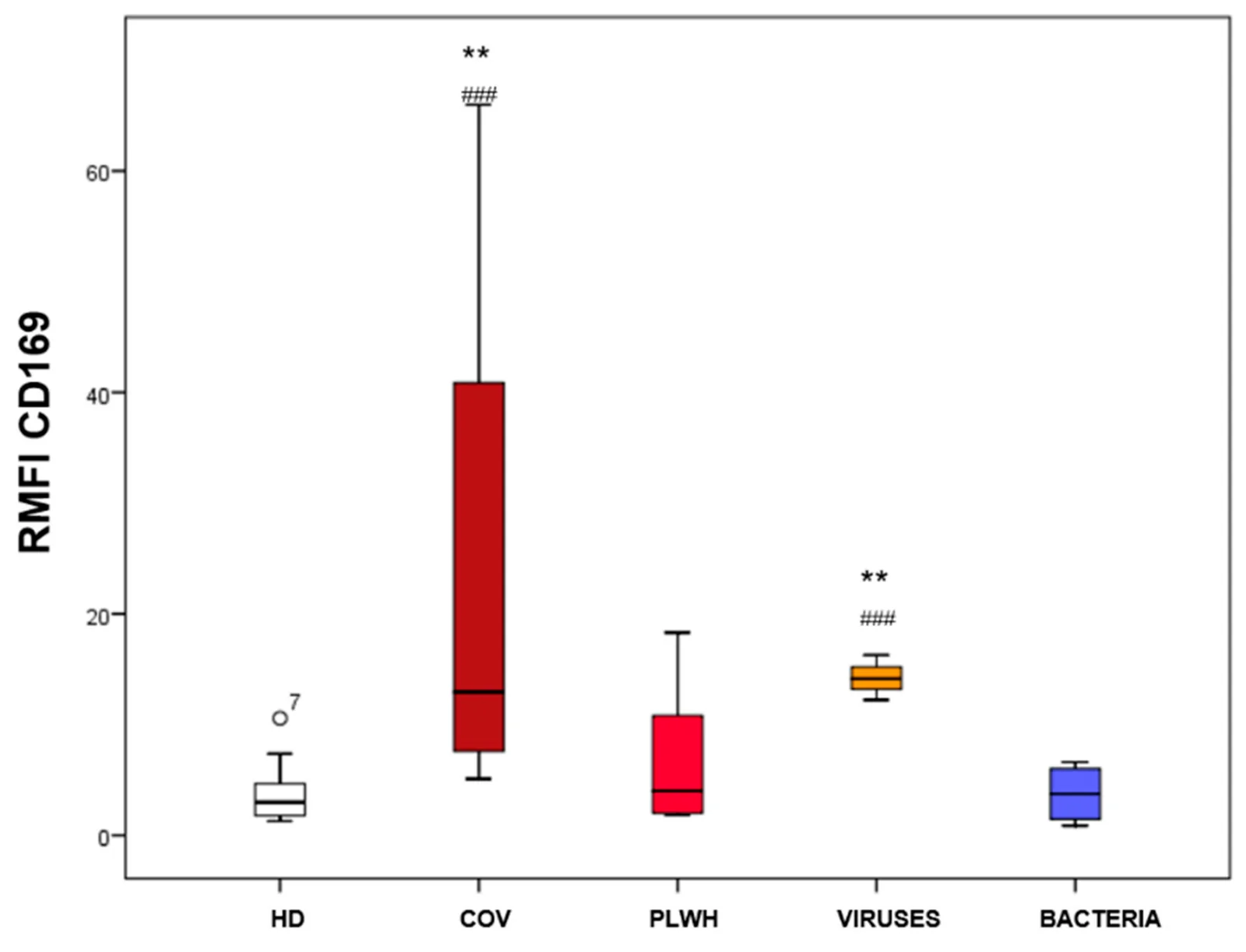
CD169 expression on HLA-DR^+^ cells assessed by relative mean fluorescence intensity (RMFI). Box-and-whisker plots showing CD169 RMFI, calculated as the ratio of the mean fluorescence intensity (MFI) of HLA-DR^+^ monocytes to that of HLA-DR^+^ lymphocytes, in healthy donors (HD), patients with COVID-19 (COV), people living with HIV (PLWH), other viral infections (VIRUS), and bacterial infections (BACTERIA). The center line represents the median, the box indicates the interquartile range (IQR), and the whiskers represent the minimum and maximum values. Individual data points correspond to outliers. Horizontal bars indicate statistically significant comparisons between groups. Statistical significance was determined using Mann–Whitney U test. Infected vs. HD. ** *p* < 0.01; multiple comparison ^###^ *p* < 0.001.

CD169 expression was evaluated as the relative mean fluorescence intensity (RMFI), calculated as the ratio between the mean fluorescence intensity (MFI) of HLA-DR^+^ monocytes and HLA-DR^+^ lymphocytes across the study groups, including HD, COV, PLWH, VIRUS, and BACTERIA (Figure 1). Regarding the early tracking of viral triggers and host immune disruptions, the surface expression of CD169 on monocytes provided high accuracy. Following the parameters described by Cao et al. (2026) [18], a baseline cut-off of 50% CD169-positive monocytes enabled clinicians to distinguish acute viral phases, including early HIV-1 infection, from uninfected cohorts, with a reported detection rate of 92%. Moreover, this finding was consistent with the clinical utility of CD169 ratios for characterizing severe immunophenotypic alterations and acute oxygen requirements in viral infections such as COVID-19 [19].

In our cohort, HD exhibited low and homogeneous CD169 RMFI values, consistent with physiological levels of monocyte activation. In contrast, patients with COVID-19 showed a significant increase in CD169 RMFI compared with healthy controls (*p* < 0.01), indicating enhanced CD169 expression on HLA-DR^+^ monocytes. Similarly, patients with other viral infections displayed elevated CD169 RMFI values, although with greater interindividual variability, and several individuals exhibited markedly increased expression. Patients with HIV infection showed intermediate CD169 RMFI values, generally higher than those observed in healthy donors but lower than those detected in the COVID-19 and other viral infection groups. Likewise, patients with bacterial infections exhibited moderate CD169 expression with lower variability than the viral infection groups. This total absence of monocyte CD169 induction during bacterial insults underscored its high specificity as a diagnostic tool.

Statistical analysis revealed significantly higher CD169 RMFI values in both the COVID-19 and other viral infection groups compared with healthy donors (both *p* < 0.01). No significant differences were observed between the remaining study groups.

Overall, these findings demonstrate that CD169 expression was preferentially upregulated during acute viral infections, particularly SARS-CoV-2 infection, supporting its role as a biomarker of type I interferon-driven monocyte activation and innate immune response.

### 3.7. CD64 Expression on HLA-DR^+^ Cells Assessed by Relative Mean Fluorescence Intensity (RMFI)

**Figure 2:**
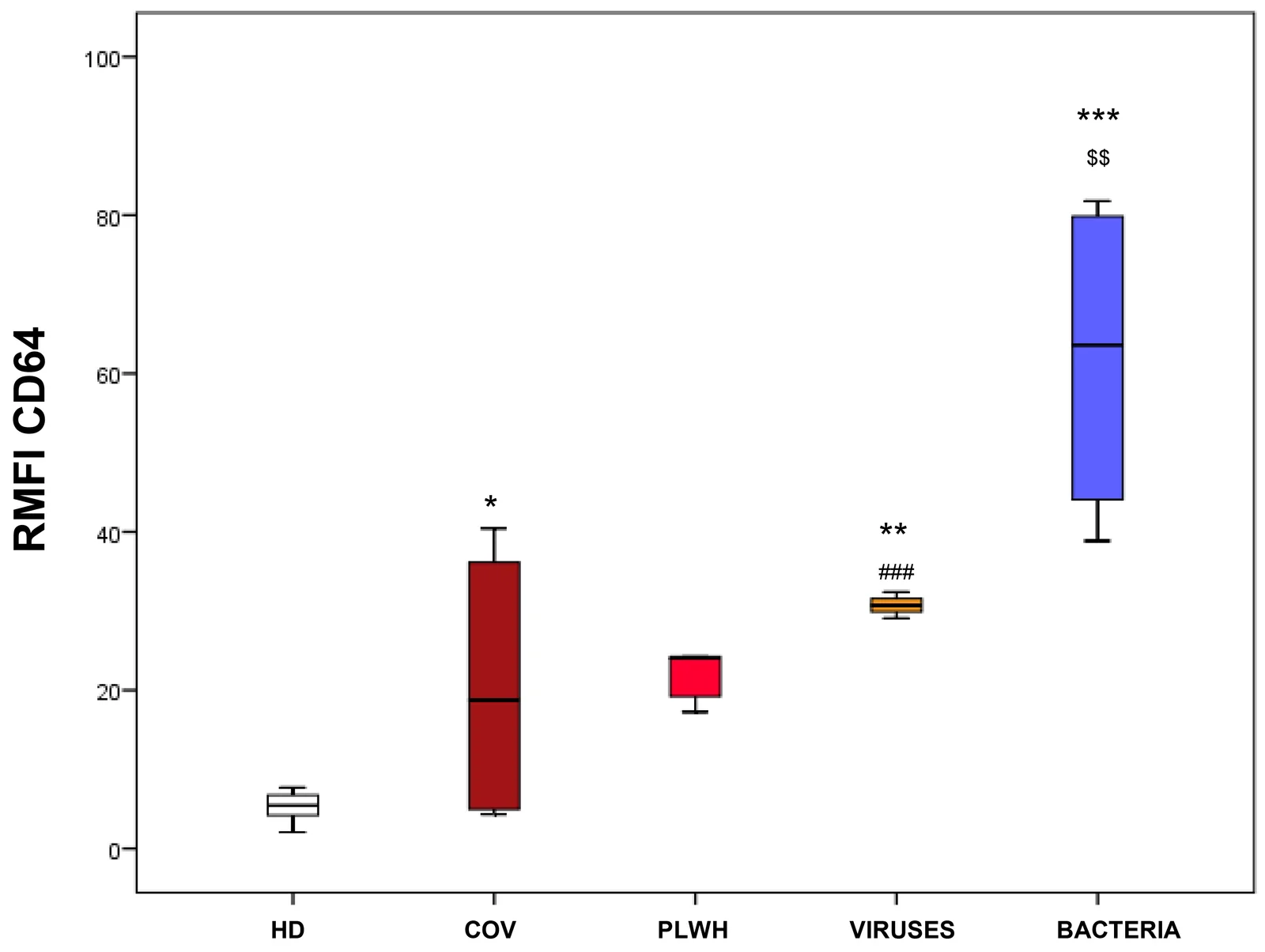
CD64 expression on HLA-DR^+^ cells assessed by relative mean fluorescence intensity (RMFI). Box-and-whisker plots showing CD64 RMFI profiles in Healthy Donors (HD), patients with COVID-19 (COV), People Living with HIV (PLWH), patients with other viral infections (VIRUSES), and patients with bacterial infections (BACTERIA). The horizontal line within each box defines the median; box margins indicate the interquartile range (IQR), and whiskers define the overall data distribution boundaries (minimum and maximum values). Individual data points plotted outside whiskers represent clinical outliers, including a high-level reactive outlier marked in the PLWH cohort. Statistical significance was determined using the using Mann–Whitney U test. * *p* < 0.05, ** *p* < 0.01, and *** *p* < 0.001 versus healthy donors (HD); ^###^ *p* < 0.001 versus the COVID-19 group; ^$$^ *p* < 0.01 versus all other infection groups (COVID-19, PLWH, and VIRUSES).

To fully resolve the complementary host-response landscape of our cohort, RMFI of cell-surface CD64 was systematically mapped across five matching clinical arms: HD (n = 19), COV (n = 4), PLWH (n = 5), VIRUSES (n = 3), and BACTERIA (n = 4) (Figure 2). As validated by van de Ven et al. (2023) [20], when utilized in emergency or acute care settings, nCD64 exhibits an ROC-AUC of 0.71 (95% CI: 0.64–0.79). Moreover, to achieve maximum clinical certainty as a “rule-in” test for bacterial infections, a specific MFI cut-off point of 9.4 AU yielded a specificity of 1.00 and a Positive Predictive Value (PPV) of 1.00 (with a sensitivity of 0.27 and an NPV of 0.64). This reinforces the clinical perspective of nCD64 as an indispensable biomarker for risk stratification, acute inflammation monitoring, and early detection of sepsis or acute localized complications, in line with modern literature [21,22].

In our cohort homeostatic restriction of CD64 expression was highly preserved in the Healthy Donors group, which exhibited minimal interindividual variability and baseline values tightly locked below 10 RMFI, consistent with physiological baseline conditions. In contrast, all infected patient cohorts displayed an upward shift in CD64 expression compared to healthy controls, though to fundamentally different magnitudes. Mild to moderate up-regulation was noted across the viral configurations.

The COV cohort presented a wide, heterogeneous distribution tracking up to 40 RMFI (median ~19), yet demonstrating a statistically significant increase compared to HD. The PLWH group displayed a restricted core distribution (median ~22) marked by a distinctive high-level hyper-reactive outlier at approximately 48 RMFI.

Patients within the non-COV viral arm (VIRUSES) demonstrated a narrow, uniform intermediate plateau centered at 30 RMFI, which was significantly higher than the levels observed in the COV group.

A structurally defining paradigm was observed in the Bacterial Infections cohort, which yielded a profound, high-amplitude up-regulation of CD64. The interquartile range (IQR) for the bacterial group shifted entirely above all viral medians, spanning from 44 to 80 RMFI, with a prominent cohort median of 64. CD64 expression in this bacterial arm was substantially elevated compared to HD and remained robustly segregated from all other active infection branches (COV, PLWH, and VIRUSES).

### 3.8. Comparative Analysis of Group Phenotypes

To fulfill rigorous reporting standards for small sample sizes and minimize reliance on dense symbolic annotations, standard binary testing was accompanied by non-parametric effect size estimations via Cliff’s delta (d) calculations (Table 6). Regarding RMFI CD169 expression, HD maintained a strict homeostatic baseline below 10 RMFI. In contrast, acute viral insults triggered a rapid, high-amplitude up-regulation. The COVID-19 cohort achieved the most prominent expansion (median RMFI ~19), yielding a significant divergence from baseline (U = 6.000, *p* = 0.040) with a large effect magnitude (d = 0.824). The “Other Viruses” branch showed an intermediate up-regulation plateau (median RMFI ~30; *p* = 0.099, d = 0.412, medium effect). Conversely, chronic viral conditions (PLWH; d = 0.176) and Bacterial Infections (d = 0.147) remained flat and baseline-bound, showing negligible to small effect sizes against controls.

Regarding RMFI CD64 kinetics, a complementary operational paradigm was observed. The Bacterial Infections cohort yielded a profound activation spike (median = 64, IQR 44–80 RMFI), achieving distinct significance at the cohort boundary (U = 13.000, *p* = 0.050, d = 0.618, large effect) against HD. Marked secondary up-regulations were also detected across viral configurations: the “Other Viruses” arm clustered around an intermediate plateau (U = 5.000, *p* = 0.006, d = 0.804), the PLWH branch presented an elevated core with a prominent outlier at 48 RMFI (U = 9.000, *p* = 0.006, d = 0.735), and the COVID-19 group tracked a wide expansion up to 40 RMFI (median ~19, d = 0.618). Ultimately, pairing the viral-specific fidelity of monocyte CD169 with the high-amplitude bacterial responsiveness of cell-surface CD64 provides a statistically robust, reciprocal diagnostic matrix to differentiate infectious etiologies in acute clinical triage.

### 3.9. Etiology-Specific Correlation Networks of CD169 and CD64 in Infectious Diseases

**Figure 3:**
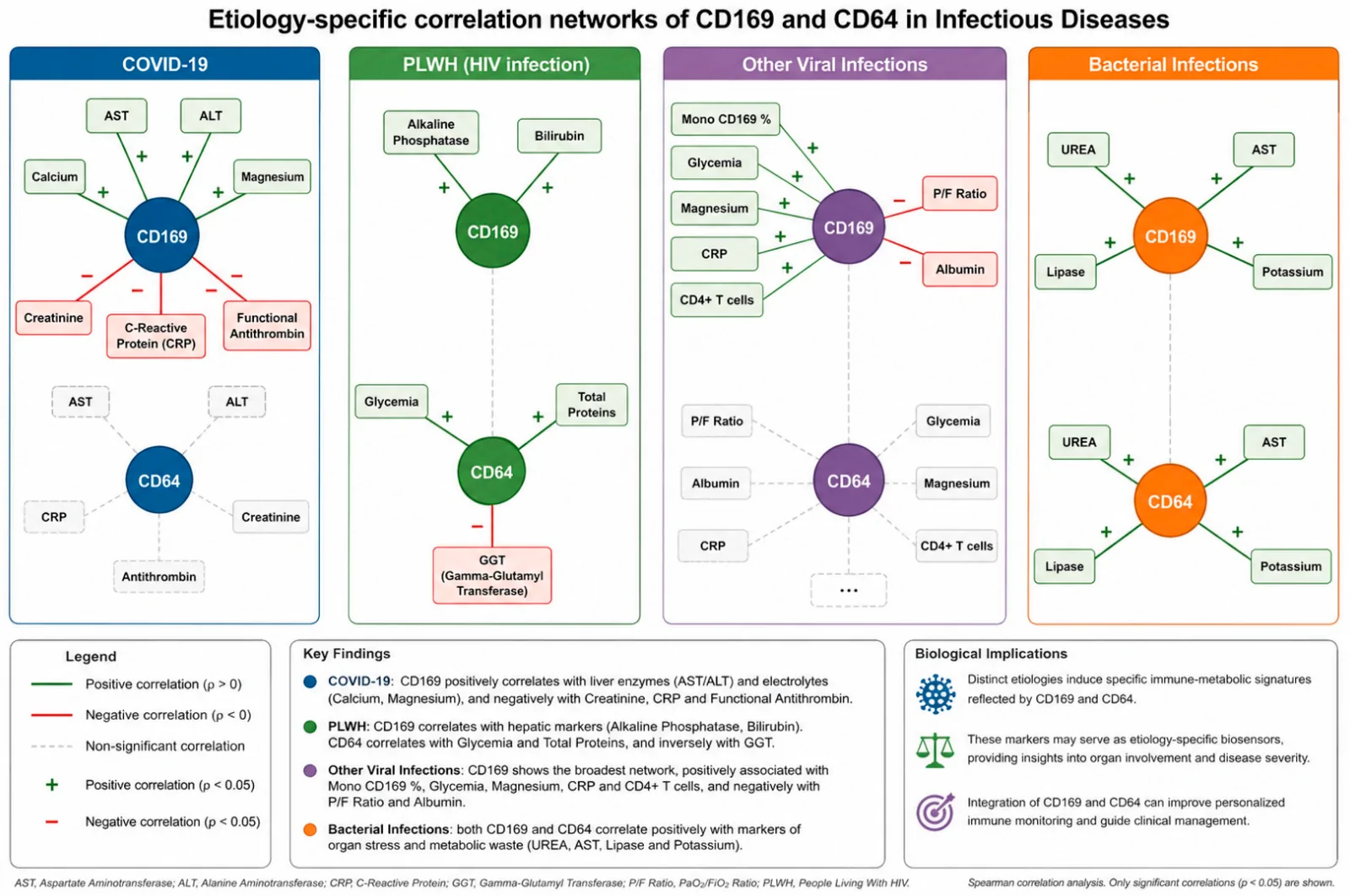
Etiology-specific correlation networks of CD169 and CD64 in infectious diseases. Schematic representation of the significant Spearman correlations between CD169 and CD64 RMFI and clinical, biochemical, and immunological parameters in COV, PLWH, other viral infections, and bacterial infections. Green lines indicate positive correlations, and red lines indicate negative correlations. Only statistically significant associations are shown (*p* < 0.05). Abbreviations: RMFI, Relative Mean Fluorescence Intensity; PLWH, people living with HIV; CRP, C-reactive protein; GGT, gamma-glutamyl transferase; P/F ratio, PaO_2_/FiO_2_ ratio. The figure was generated using Microsoft 365 Copilot 2.20260914.59.0.

Exploratory correlation analyses were performed to investigate potential associations between the immunological markers CD169 and CD64 and selected clinical and biochemical parameters across the different infectious conditions (Figure 3 and Table S3). Given the very small sample sizes of the individual subgroups, particularly the COVID-19 group, these analyses should be considered hypothesis-generating only and should not be interpreted as evidence of robust or generalizable associations.

Despite these limitations, distinct correlation patterns were descriptively observed across the different infectious conditions. In the COVID-19 group, CD169 showed positive correlations with liver enzymes (AST and ALT) and electrolytes, including calcium and magnesium, whereas inverse correlations were observed with creatinine, CRP, and antithrombin activity. Given the limited number of patients, these observations should be interpreted cautiously and require validation in larger cohorts.

In the PLWH cohort, CD169 showed positive associations with selected hepatic parameters, including alkaline phosphatase and bilirubin, whereas CD64 was associated with glycemia and total proteins and inversely correlated with GGT. These findings may suggest differences in the clinical and metabolic correlates of CD169 and CD64 in patients with HIV; however, no conclusions regarding potential protective or mechanistic effects can be drawn from these exploratory analyses.

The “Other Viral Infections” cohort displayed a broader range of exploratory associations involving CD169, including positive correlations with monocytic CD169 expression, glycemia, magnesium, CRP, and CD4^+^ T-cell counts, as well as inverse correlations with the P/F ratio and albumin. These observations may indicate potential relationships between CD169 expression and different clinical or immunological parameters across non-COVID viral infections but require confirmation in adequately powered studies.

Similarly, in the bacterial cohort, both CD169 and CD64 showed positive exploratory associations with parameters related to organ function and metabolic status, including urea, AST, lipase, and potassium. However, the small sample size precludes reliable inference regarding the consistency or biological significance of these associations.

Overall, these exploratory analyses suggest that the relationships between CD169, CD64, and clinical or biochemical parameters may differ according to the underlying infectious condition. However, because correlation analyses within these very small subgroups are statistically underpowered and highly sensitive to individual observations, the results should be interpreted exclusively as hypothesis-generating. Therefore, these findings should be validated in larger, independently recruited cohorts before considering any potential clinical or pathophysiological implications.

## 4. Discussion

Respiratory infections and sepsis represented significant clinical challenges. These difficulties were complicated by pathogen heterogeneity and rapid infection kinetics. Consequently, there was an urgent need for timely diagnostic identification. Within the framework of the national multicenter SIS-NET project, this study characterized the immunological and inflammatory landscape of patients hospitalized with severe respiratory infections.

The patients with chronic HBV infection and those with metastatic adenocarcinoma were excluded because a single individual represented each condition. Moreover, one patient with viral and bacterial coinfections were also excluded. Their exclusion avoided the generation of underpowered comparison groups. This step improved the overall robustness of subsequent subgroup analyses.

Our data showed persistent systemic inflammatory activation with etiology-specific patterns. Elevated CRP and fibrinogen levels, particularly in SARS-CoV-2 and HIV-positive patients, reflected acute cytokine storms or chronic immune activation [23]. This fibrinogen elevation correlated with systemic prothrombotic states, often termed “thrombo-inflammation” [24]. This phenomenon exacerbated endothelial damage and microvascular complications. Furthermore, markers of cellular damage, such as LDH, were elevated in acute viral and bacterial respiratory infections [25]. This indicated high cellular turnover and potential parenchymal injury. Hepatic involvement, marked by elevated ALT, was more pronounced in the SARS-CoV-2 cohort. This outcome likely resulted from direct viral tropism via ACE2 or systemic inflammatory toxicity [26].

Our findings highlighted the clinical utility of monitoring CD169 and CD64 expression on circulating myeloid cells for differential diagnosis. CD169 (Siglec-1) represented a Type I interferon-inducible protein [11]. In response to viral infections, viral nucleic acids triggered the production of Type I interferons. This process activated the JAK/STAT pathway and the subsequent transcription of the *SIGLEC1* gene [27]. Functionally, CD169 acted as a tethering receptor for viral envelope glycoproteins, facilitating antigen presentation and viral clearance. Its upregulation, quantified by the relative mean fluorescence intensity (RMFI) ratio, served as a robust signature of the viral immune response [19,28].

Conversely, CD64 (FcγRI) acted as a marker for bacterial insult. Its rapid upregulation was triggered by bacterial endotoxins or IFN-γ, which enhanced leukocyte bactericidal activity [13,27,28]. Neutrophil CD64 functioned as a robust, high-amplitude operational marker. Consistent with previous evidence, neutrophil CD64 expression was markedly increased in the BACTERIA cohort. This increase clearly separated these patients from healthy donors and all viral groups. In contrast, COVID-19 patients and people living with HIV (PLWH) showed only mild-to-moderate, heterogeneous increases. Non-COV viral infections displayed intermediate values. The substantial and non-overlapping increase observed in bacterial infections supported the diagnostic value of neutrophil CD64. These findings were consistent with previous clinical benchmarks identifying elevated neutrophil CD64 expression as a useful diagnostic tool [20].

The biomarker distribution observed in our study provided further insight into host-response immunophenotyping. As shown in Figure 3, monocyte CD169 expression was markedly increased in the acute viral cohorts (COV and VIRUSES). Conversely, the BACTERIA group displayed values comparable to those observed in healthy donors. This pattern aligned with the biological role of CD169 as an interferon-inducible marker driven by type I interferon signaling. In contrast, the different innate immune pathways activated during bacterial infections accounted for the limited CD169 induction. From a clinical perspective, the complementary assessment of monocyte CD169 and neutrophil CD64 represented a useful approach. The distinct expression patterns—high CD169/low CD64 for viral infections and low CD169/high CD64 for bacterial etiologies—provided a diagnostic framework to guide targeted therapy. Such a dual-biomarker strategy could improve diagnostic accuracy and contribute to a more appropriate use of antimicrobial therapy.

Indeed, the markers participated in distinct, etiology-specific correlation networks. In bacterial infections, robust positive associations existed between both markers and metabolic or tissue-damage markers, such as urea, AST, and lipase. This suggested that CD169 and CD64 effectively mirrored the systemic metabolic footprint of an acute bacterial challenge. Regarding the PLWH cohort, we identified a functional divergence. In these patients, CD64 expression appeared more intimately linked to glycemic control and chronic T-cell remodeling. This finding highlighted the necessity of a dual-marker approach to comprehensively assess the metabolic-immune interplay in chronic viral conditions.

The COVID-19 cohort presented a compelling paradox. The inverse relationship between CD169 and markers of severe inflammation and coagulation (such as CRP and antithrombin) suggested a key mechanism. Specifically, a decline in CD169 expression served as a critical index of immune deregulation or regulatory failure in severe disease states. Furthermore, the broad correlation of CD169 with respiratory parameters and T-cell homeostasis in other viral infections underscored its versatility. It functioned as a useful biomarker for the early detection of virus-induced lung injury and immune imbalance.

Integrating CD169 and CD64 into routine immune monitoring provided a nuanced, personalized diagnostic framework. This approach enabled accurate assessments of disease severity independent of the specific underlying pathogen. By identifying the individual “immune footprint,” this methodology supported antimicrobial stewardship. It reduced reliance on empiric broad-spectrum antibiotics, thereby mitigating the emergence of multidrug-resistant (MDR) organisms.

To transition this diagnostic matrix into routine 24/7 acute care workflows, we addressed key operational and economic factors. First, our protocol required minimal blood volume (30 µL) and no washing steps. This compressed the turnaround time to under 45 min, which perfectly fit urgent clinical triage windows. Traditional flow cytometry was historically limited by its reliance on specialized staff and laboratory infrastructure. However, this 24/7 staffing barrier was overcome by adopting fully automated, cartridge-based clinical cytometers. These systems handled sample preparation and analysis automatically near the patient.

Economically, the direct reagent costs for this targeted panel were substantially lower than those of broad molecular assays. The true value lay in secondary savings. The use of monocyte CD169 to rule out bacterial insults, combined with neutrophil CD64 to rule in sepsis, significantly reduced inappropriate antibiotic use and shortened hospital stays. Finally, absolute intensity values often varied by instrument. To ensure generalizability across different hospitals, these values were replaced with RMFI ratios. The ratios were normalized against the patient’s own resting lymphocytes, thereby providing a robust, platform-independent clinical threshold.

This study represented a pilot phase conducted within the SIS-NET multicenter framework. Due to the limited sample size, the results were interpreted as preliminary. However, this work served as a vital proof-of-concept for the utility of monocyte surface markers. As the SIS-NET project progressed, the inclusion of a larger, more diverse patient cohort was essential to validate these findings. This expansion would allow for more robust stratification of the entire cohort and further refine the diagnostic algorithms across different clinical settings.

## 5. Conclusions

In conclusion, this pilot study provided a preliminary proof-of-concept regarding the host immune response to severe respiratory infections. The findings suggested that the response followed etiology-specific phenotypic pathways. These pathways were successfully captured through flow cytometric analysis of monocyte CD169 and neutrophil CD64 expression.

These initial findings highlighted the potential utility of surface markers over traditional soluble mediators for early patient stratification. However, they were interpreted with caution due to the limited sample size of our cohort. Extensive prospective investigations and robust external validation in larger, independent cohorts were strictly required. These future studies must confirm the diagnostic accuracy, clinical feasibility, and cost-effectiveness of this approach. Until such validation was performed, these findings were considered hypothesis-generating rather than definitive diagnostic evidence. Nonetheless, they represented an encouraging first step toward the future design of personalized management strategies in the global fight against antimicrobial resistance.

## Figures and Tables

**Table 1:** Aggregated Clinical and Demographic Characteristics of the Study Cohort.

Characteristic	All Patients (N = 19)
Age (years), Median [IQR] (Range)	63.0 [50.0–71.5] (31–81)
Sex (Male), n (%)	11 (57.9%)
Charlson Comorbidity Index, Mean ± SD	3.68 ± 2.56
Comorbidities, n (%)	
—Arterial Hypertension	5 (26.3%)
—HIV Infection/Severe Immunosuppression	5 (26.3%)
—Chronic Respiratory Disease (COPD/Asthma)	3 (15.8%)
—Chronic Heart Disease/Heart Failure	3 (15.8%)
—Diabetes Mellitus	3 (15.8%)
—Former Smoker/Active Smoking	3 (15.8%)
—Chronic HBV Infection	1 (5.3%)
—Metastatic Adenocarcinoma	1 (5.3%)
—Epilepsy	1 (5.3%)
—Obesity (Class II)	1 (5.3%)
Highest Respiratory Support, n (%)	
—None	1 (5.3%)
—Venturi Mask	13 (68.4%)
—High-Flow Nasal Cannula (HFNC)	4 (21.1%)
—Non-Invasive Ventilation (NIV)	1 (5.3%)
Baseline PaO_2_/FiO_2_ (P/F) Ratio	
—Mean ± SD	248.6 ± 51.3
—>300 (Preserved gas exchange), n (%)	2 (10.5%)
—200–300 (Mild impairment), n (%)	13 (68.4%)
—100–200 (Moderate impairment), n (%)	4 (21.1%)
—<100 (Severe failure), n (%)	0 (0.0%)
Hospital Stay (days), Median [IQR] (Range)	6.0 [4.0–9.5] (2–22)
Clinical Outcome (Discharged Alive), n (%)	19 (100.0%)

Abbreviations: COPD, chronic obstructive pulmonary disease; HBV, hepatitis B virus; HIV, human immunodeficiency virus; HFNC, high-flow nasal cannula; IVIG, intravenous immunoglobulin; MTHFR, methylenetetrahydrofolate reductase; NIV, non-invasive ventilation; NYHA, New York Heart Association; PEEP, positive end-expiratory pressure; P/F ratio, arterial oxygen partial pressure to inspired oxygen fraction (PaO_2_/FiO_2_) ratio; PS, pressure support.

**Table 2:** Microorganisms detected by the FAST syndromic molecular diagnostic assay in the enrolled patient cohort.

Patient ID	Pathogen(s) Identified	Relevant Infection/Comorbidity	Group
TSB001	SARS-CoV-2	nd	COV
TSB002	*Mycoplasma pneumoniae*	nd	BACTERIA
TSB003	Influenza A virus, *Enterobacter cloacae* complex, *Klebsiella pneumoniae*, *Pseudomonas aeruginosa*	nd	COINF
TSB004	Influenza A virus	nd	VIR
TSB005	*Haemophilus influenzae*	Chronic HBV infection	HBV
TSB006	Rhinovirus/Enterovirus	nd	VIR
TSB007	Rhinovirus/Enterovirus, *Haemophilus influenzae*, *Streptococcus pneumoniae*	HIV infection	HIV
TSB008	*Candida* spp.	HIV infection	HIV
TSB009	*Pseudomonas aeruginosa*	nd	BACTERIA
TSB010	HSV-1, *Pneumocystis jirovecii*	HIV infection, Cytomegalovirus (CMV) infection	HIV
TSB011	Parainfluenza virus, Rhinovirus, Enterovirus, Adenovirus	HIV infection	HIV
TSB012	*Streptococcus pneumoniae*	nd	BACTERIA
TSB013	SARS-CoV-2	nd	COV
TSB014	*Pneumocystis jirovecii*	HIV infection	HIV
TSB015	Influenza A virus	nd	VIR
TSB016	SARS-CoV-2	nd	COV
TSB017	*Staphylococcus aureus*	Bacteremia	BACTERIA
TSB018	SARS-CoV-2	nd	COV
TSB019	Influenza A virus, *Streptococcus pneumoniae*	Metastatic adenocarcinoma	Adenocarcinoma

Abbreviations: CMV, cytomegalovirus; FAST, Fast Automated Syndromic Test; HIV, human immunodeficiency virus; HSV-1, herpes simplex virus type 1; spp., species. SARS-CoV-2 (COV = 4); Viral infection (VIR = 3); Bacterial Infection (BACTERIA = 4); people living with HIV (PLWH = 5); hepatitis B virus (HBV = 1); Adenocarcinoma (n = 1). nd, not determined.

**Table 3:** Biochemical Parameters Related to Organ Function and Inflammatory Status. The table summarizes the main biochemical parameters reflecting hepatic, renal, and metabolic function in the enrolled patients. Reference intervals are reported in the last row. Values outside the normal reference range should be indicated with asterisk *.

Biochemical Parameter	Reference Range	COV (n = 4)	VIR (n = 3)	BACTERIA (n = 4)	PLWH (n = 5)
Glucose (mg/dL)	70–100	91.00 ± 37.68	135.33 ± 59.60	83.25 ± 12.04	87.40 ± 24.35
Urea (mg/dL)	18–55	62.47 ± 30.53	50.10 ± 20.41	31.03 ± 14.12	29.85 ± 8.06
Creatinine (mg/dL)	0.73–1.18	1.15 ± 0.46	0.76 ± 0.08	0.72 ± 0.21	0.52 ± 0.10
Albumin (g/dL)	3.50–5.20	3.96 ± 0.32	3.93 ± 0.41	3.45 ± 0.76	3.66 ± 0.78
AST (U/L)	5–34	23.50 ± 13.53	71.33 ± 37.45	98.33 ± 114.94 *	45.33 ± 20.84 *
ALT (U/L)	0–55	15.00 ± 7.53	42.00 ± 28.05	122.33 ± 123.64 *	28.25 ± 16.74 *
Alkaline Phosphatase (U/L)	40–150	71.89 ± 26.39	73.92 ± 17.46	78.22 ± 11.03 *	72.44 ± 19.56 *
Total Bilirubin (mg/dL)	≤1.20	0.42 ± 0.03	0.70 ± 0.25	0.54 ± 0.21 *	0.35 ± 0.14 *
GGT (U/L)	12–64	29.75 ± 18.39	74.00 ± 76.21	46.43 ± 14.95	29.00 ± 9.13 *
CRP (mg/L)	0–5	121.75 ± 62.55	155.90 ± 50.66	105.88 ± 99.26	80.20 ± 94.27

**Table 4:** Comparative analysis of baseline electrolyte profiles, coagulation parameters, and cellular injury biomarkers among the study groups.

Parameter	Reference Range	COV (n = 4)	VIR (n = 3)	BACTERIA (n = 4)	PLWH (n = 5)
Na (mmol/L)	136–145	139.0 ± 2.8	138.0 ± 3.0	145.5 ± 16.8 *	137.2 ± 3.6
K (mmol/L)	3.5–5.1	4.00 ± 0.37	3.43 ± 0.42 *	3.63 ± 0.15	4.37 ± 0.15
Cl (mmol/L)	98–108	103.2 ± 3.3	102.0 ± 3.6	101.7 ± 1.5	101.5 ± 2.5
Ca (mg/dL)	8.4–10.2	8.65 ± 0.06	8.93 ± 0.59	8.60 ± 0.70	8.77 ± 0.39
Mg (mg/dL)	1.6–2.6	2.08 ± 0.18	2.15 ± 0.58	2.02 ± 0.09	2.25 ± 0.31
P (mg/dL)	2.3–4.7	3.60 ± 1.09	2.90 ± 0.46	2.33 ± 0.55	3.25 ± 0.70
PT Ratio	0.8–1.3	0.74 ± 0.16 *	0.87 ± 0.14	0.84 ± 0.12	0.82 ± 0.23
INR	0.8–1.2	1.27 ± 0.22 *	1.10 ± 0.17	1.18 ± 0.15	1.24 ± 0.38 *
aPTT (s)	25–38.5	47.90 ± 2.17 *	39.97 ± 3.27 *	40.25 ± 3.75 *	43.04 ± 16.70 *
aPTT Ratio	0.8–1.2	1.40 ± 0.00 *	1.17 ± 0.15	1.12 ± 0.10	1.24 ± 0.50 *
LDH (U/L)	125–220	218.0 ± 17.3	233.0 ± 15.6 *	340.3 ± 136.4 *	363.5 ± 109.6 *
Fibrinogen (mg/dL)	200–400	493.0 ± 165.6 *	843.0 ± 280.0*	605.0 ± 211.6 *	595.4 ± 175.6 *
AT Activity	0.8–1.2	0.75 ± 0.15 *	0.98 ± 0.23	0.98 ± 0.10	1.01 ± 0.12

Values are expressed as Mean ± Standard Deviation (SD). Missing values within the patient datasets were dynamically excluded from group analysis. An asterisk (*) indicate mean values falling outside the normal clinical reference intervals. Abbreviations: AT, antithrombin activity; aPTT, activated partial thromboplastin time; Cl, chloride; Ca, calcium; INR, international normalized ratio; K, potassium; LDH, lactate dehydrogenase; Mg, magnesium; Na, sodium; P, phosphorus; PT ratio, prothrombin time ratio.

**Table 5:** Distribution of peripheral immune cell subsets in healthy donors and patients with different infectious diseases.

% of Positive Cells	HD			COV			PLWH			VIRUSES			BACTERIA		
	25	50	75	25	50	75	25	50	75	25	50	75	25	50	75
Leukocytes	82.67	88.14	94.49	95.36	97.08	98.35	98.13	98.90	99.15	94.95	96.94	98.61	96.81	98.24	99.45
Lymphocytes	5.63	11.31	33.41	8.94	23.78	35.20	9.20	17.90	46.76	7.49	11.59	21.60	20.99	22.48	24.48
Monocytes	6.35	8.52	11.86	7.20	18.88 **	29.65	15.65	19.92 **	35.79	10.56	13.46	15.29	9.78	12.69	14.69
Granulocytes	27.11	33.06	43.24	20.11	30.54	45.50	13.74	27.33	55.91	46.06	54.14	60.10	21.93	47.11	67.11
T cell (CD3^+^)	25.61	52.57	65.60	30.79	36.42	72.07	9.60	64.42	70.18	40.06	58.49	60.56	53.78	58.94	68.94
T helper (CD3^+^CD4^+^)	50.04	56.18	62.51	30.52	54.60	67.47	2.04	7.14 **	40.91	41.20	51.85	73.66	53.09	70.93	80.93
Cytotoxic T Lymphocytes (CD3^+^CD8^+^)	25.93	37.51	40.84	25.05	38.03	49.80	56.15	85.11 **	89.94	23.68	43.26	51.49	22.16	26.18	36.18
CD4^+^CD8^+^	0.00	0.00	0.00	0.56	1.62 **	1.94	0.34	1.07	1.99	0.82	2.48 **	4.87	0.83	0.88	0.95
CD4/CD8 ratio	1.24	1.54	2.20	0.61	1.69	2.65	0.02	0.08 **	0.76	0.80	1.24	3.48	1.29	2.11	3.24
B cell (CD19^+^)	9.03	17.76	21.76	8.43	34.52 **	36.38	9.80	21.40	28.32	5.72	20.62	28.01	15.04	21.75	36.24
Natural Killer CD56^+^CD3^−^	12.19	16.11	20.59	1.02	13.92 **	31.05	10.29	12.17	15.67	5.46	16.11	21.78	14.06	17.44	23.92

** indicate statistically significant differences compared with healthy donors (HD) (*p* < 0.01); Mann–Whitney U test was used. The results of Cliff’ delta analysis was reported in Supplementary Material.

**Table 6:** Non-parametric effect size analysis (Cliff’s delta) and statistical significance of CD169 and CD64 expression between active infection cohorts and healthy controls (HD).

Comparison vs. HD (n = 17)	Biomarker	Mann–Whitney U	Exact *p*-Value	Cliff’s Delta (d)	Effect Magnitude
COVID-19 (n = 4)	RMFI CD169 RMFI CD64	6.000 13.000	0.040 * 0.216	0.824 0.618	Large Large
HIV (PLWH) (n = 4)	RMFI CD169 RMFI CD64	28.000 9.000	0.283 0.006 *	0.176 0.735	Small Large
Other Viruses (n = 3)	RMFI CD169 RMFI CD64	15.000 5.000	0.099 0.006 *	0.412 0.804	Medium Large
BACT	RMFI CD169 RMFI CD64	29.000 13.000	0.698 0.050 *	0.147 0.618	Small Large

* Indicates a statistically significant difference compared to the Healthy Donor (HD) control group (*p* < 0.05), calculated using the Mann–Whitney U test.

## Data Availability

The data supporting the findings of this study are stored in the SISNET project repository. At this stage, the datasets are not publicly available, as data collection and analysis across the entire network are still ongoing. The data will be made available after completion of the network-wide analyses, subject to the SISNET data-sharing policies.

## Supplementary Materials

The following supporting information can be downloaded at: https://www.mdpi.com/article/10.3390/microorganisms14092070/s1. Supplementary Figure S1. Flow cytometry gating strategy and evaluation of CD169 and CD64 expression. Table S1. Clinical and Demographic Characteristics of the Study Cohort. Table S2. Non-parametric effect size analysis (Cliff’s delta) and statistical significance of peripheral blood lymphocyte subpopulations compared to Healthy Donors (HD). Table S3. Etiology-Specific Spearman’s Rank Correlation Coefficients (ρ) for CD169 and CD64 Expression Profiles.

## Abbreviations

ACE2	angiotensin-converting enzyme 2
ALT	alanine aminotransferase
AMR	antimicrobial resistance
APC	allophycocyanin
aPTT	activated partial thromboplastin time
AST	aspartate aminotransferase
AT	antithrombin activity
BACTERIA	patients with bacterial infections
BAL	bronchoalveolar lavage
BSA	bovine serum albumin
CCI	Charlson Comorbidity Index
CD	cluster of differentiation
CE	Conformité Européenne/European Conformity
CET2	Comitato Etico Territoriale 2
CMV	cytomegalovirus
COPD	chronic obstructive pulmonary disease
COV	COVID-19 patients
COVID-19	coronavirus disease 2019
CRP	C-reactive protein
EDTA	ethylenediaminetetraacetic acid
ESBL	extended-spectrum β-lactamase
F	French/Gauge size
FAST	Fast Automated Syndromic Test
FDR	false discovery rate
FiO_2_	fraction of inspired oxygen
FSC	forward scatter
FSC-A	forward scatter area
GGT	gamma-glutamyl transferase
GLP	Good Laboratory Practice
HBV	hepatitis B virus
HD	healthy donors
HDs	healthy donors (plural form)
HFNC	high-flow nasal cannula
HIV	human immunodeficiency virus
HLA-DR	human leukocyte antigen-DR
HSV-1	herpes simplex virus type 1
IFN	interferon
INR	international normalized ratio
IQR	interquartile range
IVD	in vitro diagnostic
IVIG	intravenous immunoglobulin
JAK/STAT	Janus kinase/signal transducer and activator of transcription
LDH	lactate dehydrogenase
M	molar concentration
MDR	multidrug resistant
MERS-CoV	Middle East respiratory syndrome coronavirus
MFI	mean fluorescence intensity
MRSA	methicillin-resistant *Staphylococcus aureus*
MTHFR	methylenetetrahydrofolate reductase
n	number of subjects in a subgroup
N	total number of subjects in a cohort
nCD64	neutrophil cluster of differentiation 64
NIV	non-invasive ventilation
NK	natural killer
NYHA	New York Heart Association
P/F ratio	arterial partial pressure of oxygen to fraction of inspired oxygen ratio (PaO_2_/FiO_2_)
PB	Pacific Blue
PE	phycoerythrin
PEEP	positive end-expiratory pressure
PLWH	people living with HIV
PPV	positive predictive value
PT	prothrombin time
PTV	Policlinico Tor Vergata
RMFI	relative mean fluorescence intensity
ROC-AUC	receiver operating characteristic—area under the curve
RSV	respiratory syncytial virus
s	seconds
SARI	severe acute respiratory infection
SARS-CoV	severe acute respiratory syndrome coronavirus
SARS-CoV-2	severe acute respiratory syndrome coronavirus 2
SD	standard deviation
SIS-NET	Severe Infections and Sepsis clinical NEtwork
SSC	side scatter
SSC-A	side scatter area
T0	baseline/time of enrollment
WHO	World Health Organization
