# Supplementary material for: Immune Profiling and Rapid Molecular Diagnostics in Severe Respiratory Infections and Sepsis: The Potential Clinical Perspective of CD169 and CD64
Source: Microorganisms. 2026 Sep 16;14(9):2070. doi: 10.3390/microorganisms14092070 (PMC13609393; doi:10.3390/microorganisms14092070)
Supplement: Supplementary file 1 [file microorganisms-14-02070-s001.zip › microorganisms-4516594-supplementary.pdf]

Supplementary S1

Gating strategy for CD169 and CD64

As reported in Figure S1: CD169 expression was evaluated by flow cytometry using a sequential gating strategy. Leukocytes were initially identified based on their forward scatter (FSC-A) and side scatter (SSC-A) characteristics and subsequently separated into granulocyte-, monocyte-, and lymphocyte-enriched populations according to their characteristic light-scattering properties. A gate was then applied to identify HLA-DR-positive (HLA-DR^+) cells, within which CD169 expression was assessed in the selected monocyte and lymphocyte populations. CD169 expression was quantified as relative mean fluorescence intensity (RMFI) by comparing the fluorescence intensity of patient samples with that of healthy donors analysed using the same gating strategy.

For CD64 analysis, leukocytes were initially identified based on FSC-A and SSC-A characteristics. Granulocyte- and lymphocyte-enriched populations were subsequently identified according to their characteristic light-scattering profiles, and CD64 expression was evaluated within these gated populations. CD64 expression was quantified as relative mean fluorescence intensity (RMFI) by comparing patient samples with healthy donors analysed under the same experimental conditions.

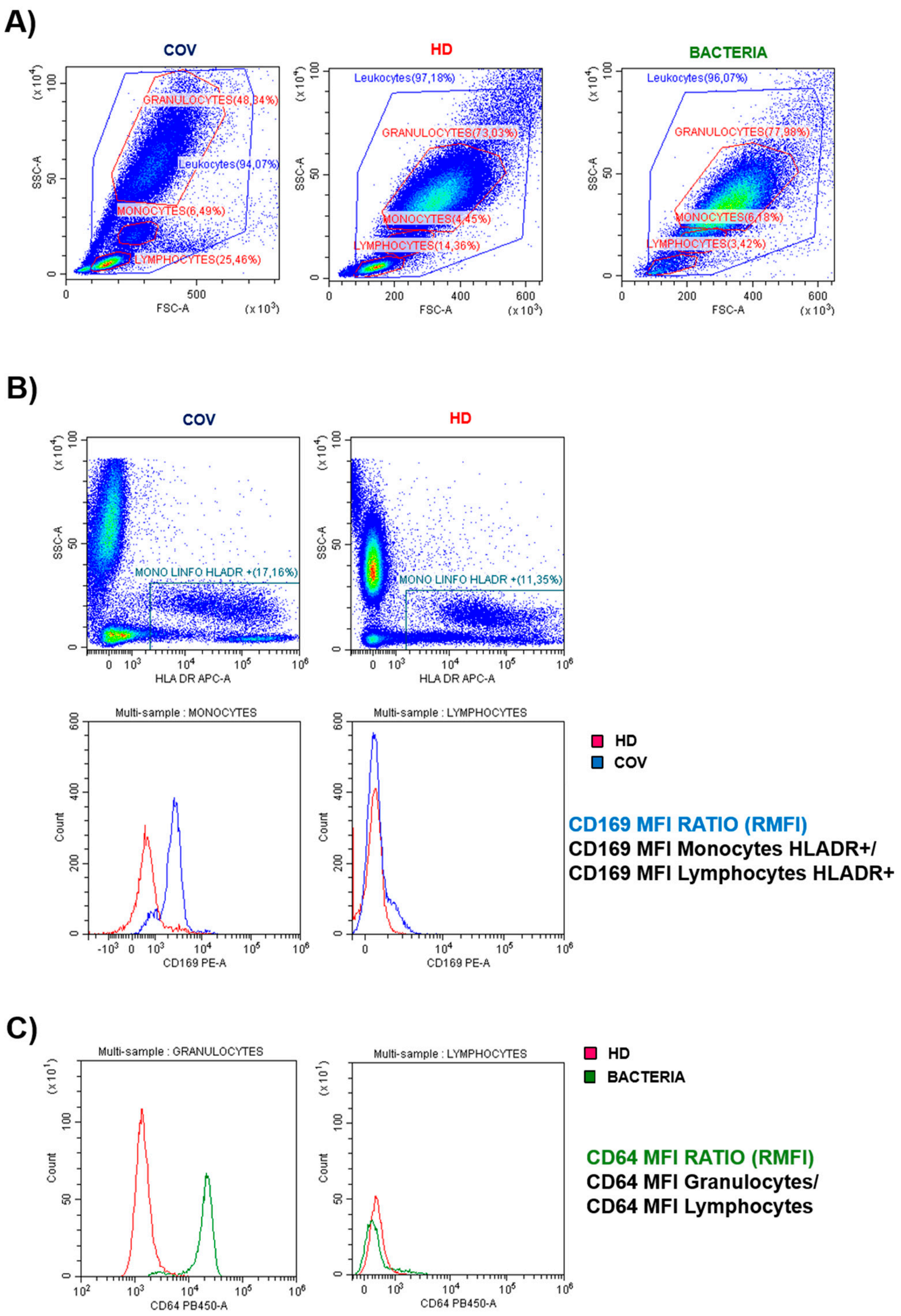

Supplementary Figure S1. Flow cytometry gating strategy and evaluation of CD169 and CD64 expression. **(A)** Representative FSC-A versus SSC-A plots showing the identification of the main leukocyte populations, including granulocytes, monocytes, and lymphocytes, in patients with COVID-19 (COV), healthy donors (HD), and patients with bacterial infections (BACTERIA). **(B)** Gating strategy for CD169 analysis. Monocyte- and lymphocyte-enriched populations were subsequently evaluated within the HLA-DR$^+$ cell population. Representative histogram overlays show CD169 expression in COV and HD samples, expressed as relative mean fluorescence intensity (RMFI). **(C)** Gating strategy for CD64 analysis. Granulocyte- and lymphocyte-enriched populations were identified according to their FSC-A/SSC-A characteristics, and CD64 expression was evaluated in the corresponding gated populations. Representative histogram overlays compare CD64 expression between HD and patients with bacterial infections (BACTERIA), expressed as RMFI.

Table S1. Clinical and Demographic Characteristics of the Study Cohort. *Demographic and clinical characteristics of the 19 patients included in the study, including age, sex, comorbidities, Charlson Comorbidity Index, highest level of respiratory support, baseline PaO_2/FiO_2 (P/F) ratio, length of hospital stay, and clinical outcome.*

Patient ID	Age (years)	Sex	Comorbidities	Charlson Comorbidity Index	Highest Respiratory Support	Baseline P/F Ratio	Hospital Stay (days)	Outcome
TSB001	71	M	Hypertension	2	None	343	5	Discharged
TSB002	61	M	Asthma, hypertension	2	Venturi mask (28%)	272	3	Discharged
TSB003	65	M	COPD, asthma	2	Venturi mask (24%)	295	2	Discharged
TSB004	79	F	Diabetes mellitus, hypertension	2	HFNC (60%, 60 L/min)	176	4	Discharged
TSB005	66	M	Chronic HBV infection	6	Venturi mask (28%)	236	6	Discharged
TSB006	56	M	COPD, major depressive disorder, class II obesity, hepatic steatosis	7	Venturi mask (35%)	276	8	Discharged
TSB007	63	F	HIV infection, smoking, severe immunosuppression, hypertension, epilepsy	8	Venturi mask (35%)	202	8	Discharged
TSB008	31	M	HIV infection, smoking, immunocompromised status, severe immunosuppression	1	Venturi mask (31%)	271	11	Discharged
TSB009	41	F	Former smoker	6	Venturi mask (28%)	333	13	Discharged
TSB010	70	F	HIV infection, chronic lung disease, COPD, former smoker, immunocompromised, primary B-cell immunodeficiency receiving monthly IVIG, MTHFR mutation	7	HFNC (40%, 60 L/min)	261	11	Discharged
TSB011	49	F	HIV infection, immunocompromised, severe immunosuppression	1	HFNC (80%, 60 L/min)	187	15	Discharged

Table S1. Clinical and Demographic Characteristics of the Study Cohort. *Demographic and clinical characteristics of the 19 patients included in the study, including age, sex, comorbidities, Charlson Comorbidity Index, highest level of respiratory support, baseline PaO_2/FiO_2 (P/F) ratio, length of hospital stay, and clinical outcome.*

Patient ID	Age (years)	Sex	Comorbidities	Charlson Comorbidity Index	Highest Respiratory Support	Baseline P/F Ratio	Hospital Stay (days)	Outcome
TSB012	52	F	Chronic heart disease	1	Venturi mask (50%)	153	7	Discharged
TSB013	72	M	Hypertension, carotid stenosis, vertebral artery stenosis, dyslipidemia	7	Venturi mask (28%)	296	3	Discharged
TSB014	47	M	HIV infection, immunocompromised, severe immunosuppression	1	Venturi mask (28%)	242	5	Discharged
TSB015	50	F	Smoking, hypertension	3	Venturi mask (28%)	250	6	Discharged
TSB016	81	F	Chronic heart disease, heart failure (NYHA class II), hypertension	1	Venturi mask (31%)	256	4	Discharged
TSB017	51	M	Former smoker	5	HFNC (50%, 60 L/min)	266	22	Discharged
TSB018	77	M	Former smoker, diabetes mellitus	2	Venturi mask (28%)	269	4	Discharged
TSB019	67	F	Metastatic adenocarcinoma	6	NIV (FiO_2 60%,	170	9	Discharged

Abbreviations: COPD, chronic obstructive pulmonary disease; HBV, hepatitis B virus; HIV, human immunodeficiency virus; HFNC, high-flow nasal cannula; IVIG, intravenous immunoglobulin; MTHFR, methylenetetrahydrofolate reductase; NIV, non-invasive ventilation; NYHA, New York Heart Association; PEEP, positive end-expiratory pressure; P/F ratio, arterial oxygen partial pressure to inspired oxygen fraction (PaO_2/FiO_2) ratio; PS, pressure support.

Supplementary S2

Non-parametric Cliff's delta (d) effect size estimations to rigorously quantify the magnitude of immunophenotypic shifts

To further delineate the host immune architecture across the different study cohorts, peripheral blood lymphocyte subpopulations — including total T cells (CD3), helper T cells (CD4), cytotoxic T cells (CD8), B cells (CD19 % Parent), and Natural Killer cells (56+3-NK % Parent), along with the computed CD4/CD8 ratio — were systematically evaluated against Healthy Donors (HD; n=17). Given the non-parametric nature of the dataset and small subgroup sample sizes, standard Mann-Whitney U test calculations were accompanied by non-parametric Cliff's delta (d) effect size estimations to rigorously quantify the magnitude of immunophenotypic shifts (Table Z).

Table S2. Non-parametric effect size analysis (Cliff's delta) and statistical significance of peripheral blood lymphocyte subpopulations compared to Healthy Donors (HD).

Comparison vs. HD (n=17)	Lymphocyte Subpopulation	Mann-Whitney U	Exact p-value	Cliff's delta (d)	Effect Magnitude
COVID-19 (n=4)	CD3	37	0.969	-0.088	Negligible
COVID-19 (n=4)	CD4	37	0.969	-0.088	Negligible
COVID-19 (n=4)	CD8	36	0.907	-0.059	Negligible
**COVID-19 (n=4)**	**CD4/CD8**	**0**	**<0.001**	**1.000**	**Large**
COVID-19 (n=4)	CD19 % Parent	18.500	0.116	0.456	Medium
COVID-19 (n=4)	56+3-NK % Parent	37.500	0.969	-0.103	Negligible
HIV (PLWH) (n=4)	CD3	46	0.945	-0.353	Medium
**HIV (PLWH) (n=4)**	**CD4**	**8**	**0.003**	**0.765**	**Large**
**HIV (PLWH) (n=4)**	**CD8**	**3**	**<0.001**	**0.912**	**Large**
**HIV (PLWH) (n=4)**	**CD4/CD8**	**0**	**<0.001**	**1.000**	**Large**
HIV (PLWH) (n=4)	CD19 % Parent	31	0.265	0.088	Negligible
HIV (PLWH) (n=4)	56+3-NK % Parent	35	0.406	-0.029	Negligible
Other Viruses (n=3)	CD3	37	0.969	-0.451	Medium
Other Viruses (n=3)	CD4	38	1.000	-0.490	Large
Other Viruses (n=3)	CD8	33	0.725	-0.294	Small
**Other Viruses (n=3)**	**CD4/CD8**	**0**	**<0.001**	**1.000**	**Large**
Other Viruses (n=3)	CD19 % Parent	31	0.611	-0.216	Small
Other Viruses (n=3)	56+3-NK % Parent	37	0.969	-0.451	Medium
Bacterial Inf. (n=4)	CD3	20	0.464	0.412	Medium
Bacterial Inf. (n=4)	CD4	15	0.226	0.559	Large
Bacterial Inf. (n=4)	CD8	23	0.651	0.324	Small
**Bacterial Inf. (n=4)**	**CD4/CD8**	**0**	**0.001**	**1.000**	**Large**
Bacterial Inf. (n=4)	CD19 % Parent	20	0.162	0.412	Medium

Bacterial (n=4)	Inf.	56+3-NK Parent	%	29	0.505	0.147	Small

Note: Statistically significant adjustments (p ≤ 0.05) and corresponding large effect magnitudes are highlighted and marked with bold text. Effect magnitudes are interpreted according to Cliff's thresholds: small (|d| ≥ 0.147), medium (|d| ≥ 0.330), and large (|d| ≥ 0.474).

The quantitative distribution of circulating lymphocyte subsets revealed highly specific immune signatures across active infection arms. A major, structurally defining paradigm was observed across all four infected cohorts (COVID-19, PLWH, Other Viruses, and Bacterial Infections), where the CD4/CD8 ratio collapsed entirely to a median of 0.000, yielding ultimate statistical significance ($p < 0.001$) and an absolute, maximum effect size magnitude (Cliff's d = 1.000) against Healthy Donors. This universal inversion highlights an acute homeostatic disruption characterized by profound T-helper depletion and/or cytotoxic T-cell expansion.

Distinctive subgroup patterns were identified outside this shared profile. In the COVID-19 cohort, individual T-cell arms remained balanced relative to controls (CD3, CD4, CD8 $p > 0.05$), suggesting that the CD4/CD8 collapse was driven by coordinated, proportional cellular shifts rather than isolated lineage loss. Conversely, the PLWH group presented severe, targeted immunophenotypic dysregulation. Beyond the inverted CD4/CD8 ratio, patients living with HIV exhibited a marked depletion of helper T cells (CD4: $U = 8.000$, $p = 0.003$, $d = 0.765$, Large effect) paired with a massive expansion of cytotoxic T cells (CD8: $U = 3.000$, $p < 0.001$, $d = 0.912$, Large effect). This classic hallmark of chronic retroviral presentation was uniquely pronounced in this arm.

In the 'Other Viruses' and 'Bacterial Infections' cohorts, total T-cell pools and peripheral B cell or NK cell fractions (CD19, 56+3-NK) demonstrated stable homeostatic maintenance without statistically significant variations against Healthy Donors ($p > 0.05$, d falling within negligible/small ranges). These findings indicate that while the CD4/CD8 ratio remains an ultra-sensitive host-response indicator for systemic immune mobilization across all major classes of acute pathogens, the selective, high-amplitude tracking of individual CD4 and CD8 counts isolates retroviral immunopathology from acute respiratory viral or bacterial insults within our patient cohort.

Supplementary S3

Table S3. Etiology-Specific Spearman's Rank Correlation Coefficients (ϱ) for CD169 and CD64 Expression Profiles

Biomarker	Clinical / Biochemical Parameter	COVID-19 (COV) (n = 4)	PLWH (HIV) (n = 5)	Other Viruses (n = 3)	Bacterial (n = 4)
RMFI CD169	AST (U/L)	**ϱ = 1.000 ($p < 0.001$)**	—	—	**ϱ = 1.000 ($p < 0.001$)**
	ALT (U/L)	**ϱ = 1.000 ($p < 0.001$)**	—	—	—
	Serum Calcium (mg/dL)	**ϱ = 1.000 ($p < 0.001$)**	—	—	—
	Serum Magnesium (mg/dL)	**ϱ = 1.000 ($p < 0.001$)**	—	**ϱ = 1.000 ($p < 0.001$)**	—
	Monocyte CD169 % Parent	—	**ϱ = 0.900 ($p = 0.037$)**	**ϱ = 1.000 ($p < 0.001$)**	—
	Blood Glucose (mg/dL)	—	—	**ϱ = 1.000 ($p < 0.001$)**	—
	C-Reactive Protein (mg/L)	**ϱ = -1.000 ($p < 0.001$)**	—	**ϱ = 1.000 ($p < 0.001$)**	—
	CD4+ T Cells	—	—	**ϱ = 1.000 ($p < 0.001$)**	—
	Alkaline Phosphatase (U/L)	—	**ϱ = 1.000 ($p < 0.001$)**	—	—
	Total Bilirubin (mg/dL)	—	**ϱ = 1.000 ($p < 0.001$)**	—	—
	Blood Urea (mg/dL)	—	—	—	**ϱ = 1.000 ($p < 0.001$)**
	Lipase (U/L)	**ϱ = 1.000 ($p < 0.001$)**	—	—	**ϱ = 1.000 ($p < 0.001$)**
	Serum Potassium (mmol/L)	—	—	—	**ϱ = 1.000 ($p < 0.001$)**
	Serum Creatinine (mg/dL)	**ϱ = -1.000 ($p < 0.001$)**	—	—	—
	Functional Antithrombin	**ϱ = -1.000 ($p < 0.001$)**	—	—	—

	PaO_2/FiO_2 (P/F) Ratio	—	—	**$\varrho = -1.000$ ($p < 0.001$)**	—
	Serum Albumin (g/dL)	—	—	**$\varrho = -1.000$ ($p < 0.001$)**	—
RMFI CD64	Blood Glucose (mg/dL)	—	**$\varrho = 1.000$ ($p < 0.001$)**	—	—
	Total Proteins (g/L)	**$\varrho = 1.000$ ($p < 0.001$)**	**$\varrho = 1.000$ ($p < 0.001$)**	—	—
	Blood Urea (mg/dL)	—	—	—	**$\varrho = 1.000$ ($p < 0.001$)**
	AST (U/L)	—	—	—	**$\varrho = 1.000$ ($p < 0.001$)**
	Lipase (U/L)	**$\varrho = 1.000$ ($p < 0.001$)**	—	—	**$\varrho = 1.000$ ($p < 0.001$)**
	Serum Potassium (mmol/L)	—	—	—	**$\varrho = 1.000$ ($p < 0.001$)**
	GGT (U/L)	—	**$\varrho = -1.000$ ($p < 0.001$)**	—	—

Note: Data are presented as Spearman's rank correlation coefficient (ρ) followed by the unadjusted p-value in parentheses. Only statistically significant associations or maximum absolute correlations ($\rho = \pm 1.000$) matching the etiology-specific networks in Figure 3 are filled. Non-significant relationships are denoted by em-dashes (—). Abbreviations: RMFI, Relative Mean Fluorescence Intensity; COV, COVID-19; PLWH, people living with HIV; GGT, gamma-glutamyl transferase; AST, aspartate aminotransferase; ALT, alanine aminotransferase.
Due to the very limited sample size within individual patient subgroups, these correlation analyses have an extremely low numerical power. Consequently, all observed associations must be interpreted strictly as exploratory and hypothesis-generating preliminary findings, rather than robust clinical or mechanistic evidence.
